# Modified Method of Contrast Transthoracic Echocardiography for the Diagnosis of Patent Foramen Ovale

**DOI:** 10.1155/2019/9828539

**Published:** 2019-05-09

**Authors:** Enfa Zhao, Yajuan Du, Hang Xie, Yushun Zhang

**Affiliations:** Department of Structural Heart Disease, the First Affiliated Hospital of Xi'an Jiaotong University, Xi'an 710061, China

## Abstract

**Purpose:**

To compare the sensitivity and specificity of modified and traditional methods of contrast echocardiography of the right portion of the heart in patients with a suspicion of patent foramen ovale (PFO).

**Methods:**

The study population consisted of 506 patients with high clinical suspicion of PFO. The traditional Valsalva maneuver consists of expiration against a closed glottis after a full inspiration. A modified Valsalva maneuver was performed with a handmade pressure monitoring device, which measured pressure during performance of the Valsalva maneuver. Modified and traditional methods of contrast echocardiography were performed among all patients. Contrast transesophageal echocardiography (TEE) was regarded as the gold standard.

**Results:**

A total of 279 patients with PFO were confirmed by TEE. 259 cases (sensitivity: 92.83%) were detected by a modified method of contrast echocardiography of the right portion of the heart, while 234 cases were detected using the traditional method (sensitivity: 83.87%). The sensitivity of modified contrast echocardiography of the right portion of the heart was significantly higher than that of the traditional method (92.83% vs. 83.87%,* P*=0.001). However, there was no significant difference in the specificity of the two methods for the diagnosis of PFO (97.35% vs. 96.03%,* P*=0.431). Additionally, the results of semiquantitative evaluation of PFO using modified method failed to show a more positive rate than shown by the traditional method (Z=−1.782,* P*=0.075).

**Conclusions:**

Modified contrast echocardiography of the right portion of the heart yielded a higher sensitivity than the traditional method, which contributed to the diagnosis of cardiac PFO. The research was a part of a register study (https://register.clinicaltrials.gov/ ClinicalTrials ID: NCT02777359).

## 1. Introduction

A patent foramen ovale (PFO) is characterized by a lap-like opening in the atrial septum secundum after birth, which is present in approximately 25% of adults[1]. The presence of a PFO has been reported to be strongly associated with a number of disease processes, including cryptogenic stroke, transient ischemic attack (TIA), migraine headaches, peripheral arterial embolism, platypnea-orthodeoxia syndrome, and decompression sickness[2–5]. Therefore, the identification of PFO in these patients is of great significance. At present, commonly used examinations of PFO right-to-left shunt (RLS) are based on three different modalities. Currently, contrast transesophageal echocardiography (c-TEE) is widely accepted as the gold standard for PFO diagnosis[6]. However, TEE is a semi-invasive, tedious, and complex diagnostic tool, which may result in discomfort and stress during the process. The diagnostic sensitivity of PFO-RLS by contrast transcranial Doppler (c-TCD) is similar to that of c-TEE[7, 8]. However, c-TCD has a limited ability to differentiate cardiac from pulmonary RLS and to provide a sufficient temporal bone window in elderly patients[9, 10]. Currently, contrast transthoracic echocardiography (c-TTE) with the Valsalva maneuver is extensively used for the detection and semiquantitative assessment of PFO-RLS. The original maneuver was reported by Valsalva in 1704 and consists of expiration against a closed glottis after a full inspiration. Today, the Valsalva maneuver is widely used by physicians in daily clinical practice[11–14]. However, the traditional Valsalva maneuver is performed by expiration against a closed nose and mouth[15, 16]. Several studies have recently reported the clinical practice use of a modified Valsalva maneuver, which use a device to monitor the effectiveness of the Valsalva maneuver[12, 17–22]. At present, there are few studies comparing diagnostic sensitivity in the traditional and modified Valsalva maneuvers. Thus, the aim of this research was to compare the sensitivity and specificity of the traditional and modified Valsalva maneuvers in the detection of PFO-RLS when c-TEE was used as a gold standard.

## 2. Materials and Methods

### 2.1. Patient Population

We prospectively investigated 506 consecutive patients (192 men, 314 women; mean age, 41.49±13.34 years) admitted to the department of structural heart disease in our hospital from September 2016 to June 2018 who suffered from TIA, cryptogenic stroke, migraine headaches, and cerebral infarction of unknown cause. Patients with poor image quality or who are unable to perform the Valsalva maneuver were excluded from this study. All patients or their relatives provided written informed consent to participate in this study prior to the examination. The study protocol was approved by the ethics committee of the First Affiliated Hospital of Xi'an Jiaotong University (No: XJTU1AF2015LSL-049) and was performed in accordance with the CONSORT 2010 guidelines. Baseline characteristics of included patients are summarized in Table 1.

### 2.2. TTE Imaging and TEE Examination

**Figure 1:**
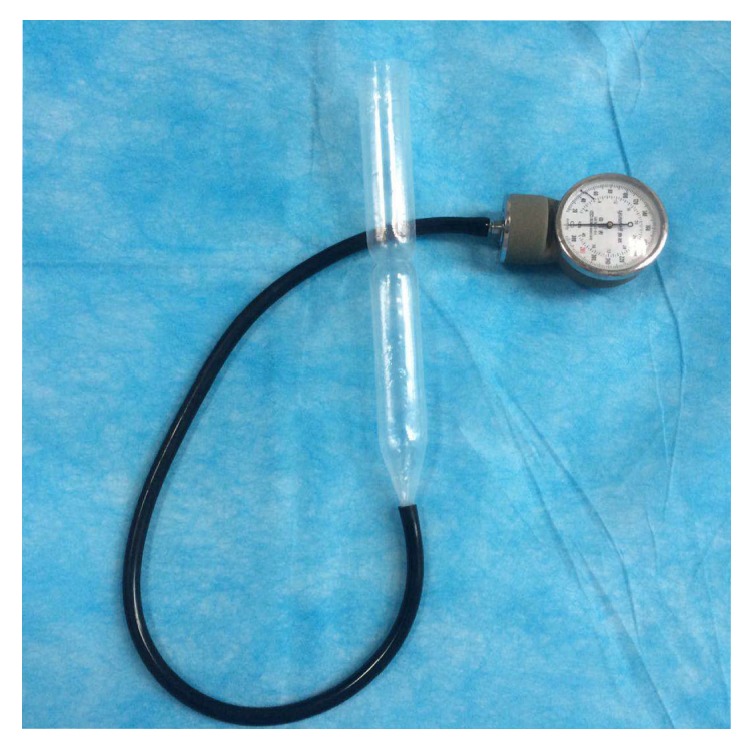
Handmade pressure monitoring device.

Contrast-TTE was conducted using the GE Vivid E9 platform equipped with a 3.7–5 MHz M5S transducer (Horten, Norway). All patients were asked to keep still in the left lateral position and were trained to breathe calmly. Conventional echocardiography was carried out to acquire the standard apical, parasternal, and subxyphoid four-chamber views. Color flow Doppler was used to observe whether there was RLS at the foramen ovale of the interatrial septum. The handmade pressure monitoring device used in this study is shown in Figure 1. After the sphygmomanometer cuff was removed, a disposable plastic tube was connected with the rubber pipe of the manometer. In this way, pressure could be monitored when patients performed the Valsalva maneuver. The injectable contrast agent was a mixture of 1mL of air, 8 mL of saline solution, and 1 mL of patient blood. The Valsalva maneuver was considered effective when a minimum reading of 40 mmHg on the manometer was observed[23]. The contrast agent was intensively mixed back and forth 20 times between two 10-mL syringes connected by a three-way stopcock. The medium was quickly injected via the established route of the anterior elbow vein. The procedure was randomly performed by first using either the traditional Valsalva maneuver or the modified Valsalva maneuver. There was an interval of at least 10 minutes between the two methods. Patients were also instructed to perform an effective Valsalva maneuver before the tests in order to reach and maintain a pressure of 40 mmHg for at least 5 seconds[19, 24, 25]. This was performed by blowing into the plastic pipe connected to the manometer device. Following this, all patients were instructed to exhale quickly, at which point sequence microbubbles were observed in the left atrium. If microbubbles appeared in the left atrium within three cardiac cycles after release, RLS was considered to be derived from a PFO. If microbubbles appeared in the left atrium after more than three cardiac cycles, RLS was assumed to originate from a pulmonary arteriovenous malformation[26]. RLS was graded according to the highest number of microbubbles observed in the left chamber in a single frame: image-negative (no microbubbles), small (1-10 microbubbles), moderate (11-30 microbubbles), or extensive (≥30 microbubbles or left chamber opacification)[27]. All procedures were recorded using both the traditional and modified methods. All findings were further analyzed retrospectively by an experienced sonographer who was blinded to the methods used.

**Figure 2:**
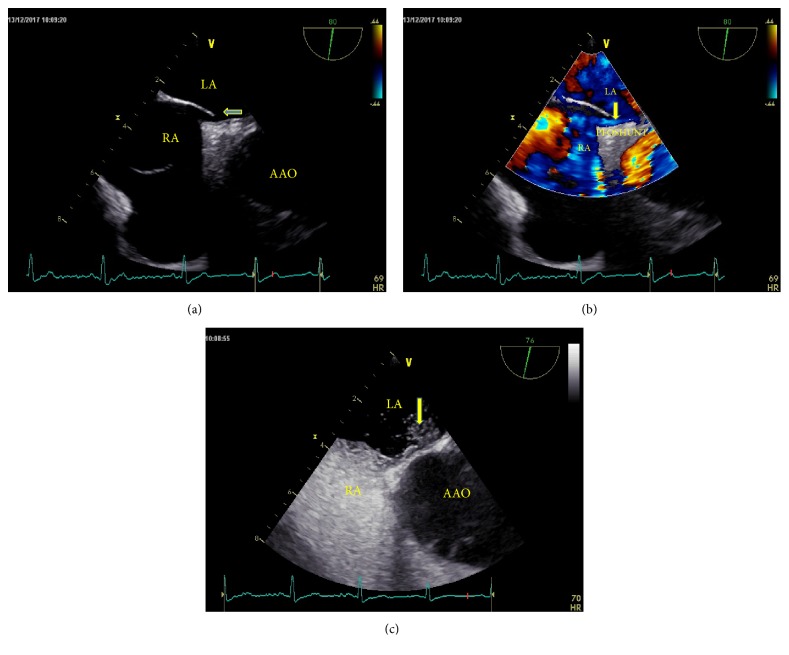
(a) Two-dimensional TEE displaying a “slit-like” channel between the left and right atria, revealing the presence of a PFO. (b) Doppler color flow imaging showing spontaneous PFO-left-to-right shunt. (c) TEE bubble study showing the microbubbles passing through the PFO.

TEE was performed using the GE Vivid E9 platform fitted with a 2.9–8MHz multifrequency probe. The TEE procedure was conducted according to the methods described in our previous study[14]. PFO-RLS was confirmed both in two-dimensional and color Doppler ultrasonography (Figures 2(a) and 2(b)). A TEE bubble study was performed as needed to confirm the presence of a PFO (Figure 2(c)).

### 2.3. Statistical Analysis

Qualitative variables were presented as percentages and continuous data were expressed as the mean ± standard deviation. A chi-square test was used to compare the sensitivity and specificity between the two methods. Semiquantitative shunt grading between the two methods was compared using the Wilcoxon-Mann-Whitney test. A* P* value of <0.05 indicated statistical significance. All data were analyzed using SPSS software (version 18.0.1, SPSS Inc.).

## 3. Results

### 3.1. Sensitivity and Specificity between the Different Methods

A total of 506 patients (314 females, median age 41.49±13.34 years) with a high clinical suspicion of PFO were included from October 2016 to June 2018. Table 1 shows the baseline characteristics of the included patients. No patient experienced a noticeable adverse event during either procedure or during the 12-hour followup period. PFO-RLS was identified by c-TEE in 279 patients (55.13%). TTE bubble study with the traditional Valsalva maneuver identified 234 patients with PFO-RLS. TTE bubble study with a modified Valsalva maneuver identified 259 patients with RLS (Table 2). The sensitivity of the TTE bubble study of right portion of the heart with the modified method was significantly higher with the traditional method (92.83% vs. 83.87%,* P*=0.001). However, there was no significant difference in the specificity of the two methods for the diagnosis of PFO (97.35% vs. 96.03%,* P*=0.431).

### 3.2. Semiquantitative Shunt Grading of RLS

The results of semiquantitative shunt grading using the two methods are shown in Table 3. The semiquantitative evaluation of RLS using the modified method did not prove superior to the traditional method, as no statistically significant differences were found using the Wilcoxon-Mann-Whitney test (*Z*=−1.782,* P*=0.075).

## 4. Discussion

To the best of our knowledge, this is the first prospective study comparing the sensitivity and specificity of traditional and modified Valsalva maneuvers for the detection of PFO-RLS when c-TEE was used as a gold standard. Our preliminary study revealed that a TTE bubble study with a modified Valsalva maneuver yielded a higher sensitivity than that with the traditional Valsalva maneuver, without producing noticeable adverse events. However, there were no significant differences in terms of specificity or semiquantitative shunt grading for the diagnosis of PFO-RLS. Therefore, we believe that a modified Valsalva maneuver can be an effective alternative to the traditional method.

A PFO is usually hemodynamically negligible, but it is regarded as the leading cause of RLS. Increasing experimental evidence has shown that PFO is closely related to cerebral ischemia and migraine symptoms[28]. Recent randomized controlled studies have confirmed that PFO closure was associated with a lower rate of recurrent stroke than medical therapy alone among patients who suffered from cryptogenic ischemic stroke[29–31]. Therefore, the exact identification of PFO-RLS is clinically significant. Echocardiography is the mainstay of PFO-RLS diagnosis. The TTE bubble test in combination with a provocation maneuver has been widely used for PFO-RLS detection. Provocation maneuvers that increase right atrial pressure have been shown to enhance PFO-RLS detection. Previous studies have also reported that the Valsalva maneuver may increase the sensitivity of TTE[32]. Recently, a simple handmade device to monitor the effectiveness of the Valsalva maneuver has been used clinically. However, it is unclear whether this device has a significant advantage over the traditional method of PFO-RLS detection. In the present study, the handmade device was used to monitor Valsalva maneuver strain pressure, and we concluded that this modified method yielded a higher sensitivity than the traditional method although there was no significant difference with respect to specificity. Several reasons may account for this finding. Firstly, when the traditional method was used, the effectiveness of the Valsalva maneuver was tested by observing a decreased peak maximum velocity of 25% in the Doppler spectrum after the maneuver[27, 33], which was not always easy to quantitatively assess. However, when the modified method was used, a persistent pressure of 40 mmHg was ensured during the procedure, which was much easier for patients to handle and had potential advantages, including the opportunity to quantitatively assess all patient ability to conduct the maneuver. Secondly, persistent pressure was visible to both patients and the sonographer during the modified method, which ensured the effectiveness of the Valsalva maneuver. The ability of patients to perform an effective Valsalva maneuver is clinically important in PFO-RLS detection. Therefore, it is crucial to explain the maneuver in detail to patients. Thirdly, a previous study reported that 87.5% of patients generated a higher pressure with the manometer than with the traditional Valsalva maneuver when transcranial Doppler was used[22], which may also be the case when a transthoracic echocardiography bubble test is used.

Although the conclusions of this preliminary research were novel and clinically important, there were also several limitations. The first limitation was that the order of randomization may have helped some patients perform an effective Valsalva without the device, and there may have been a practice effect. In addition, the underlying physical mechanism for this finding is still unclear, and for this reason animal research or human experimentation is urgently needed to elaborate this potential mechanism. Further prospective multicenter studies with larger populations are needed to confirm and extend the conclusions of our study.

In conclusion, the present study revealed that the modified Valsalva maneuver yielded a higher sensitivity than the traditional method in detecting PFO-RLS. Therefore, we recommend performing the TTE bubble test with a modified Valsalva maneuver with a strain monitored by a manometer. This device may be helpful in the diagnosis of patent foramen ovale in practice.

## Figures and Tables

**Table 1:** Baseline clinical characteristics of the study population.

Clinical features	N (%)
Age (year) mean ± standard deviation	41.49±13.34
Sex (male/female)	192/314
Hypertension	66/506(13.04%)
Diabetic mellitus	62/506(12.25%)
Coronary heart disease	36/506(7.11%)
Arrhythmia	16/506(3.16%)
Clinical symptoms	
Cryptogenic stroke	156/506(30.83%)
Transient ischemic attack	64/506(12.69%)
Migraine	128/506(25.29%)
Cerebral infarction	99/506(19.56%)
Loss of consciousness	39/506(7.70%)
Hypoxemia	18/506(3.55%)

**Table 2:** TEE and TTE bubble study with modified and traditional Valsalva maneuver.

c-TTE		TEE		
		PFO(n=279)	Without PFO (n=227)	In total
Traditional method	Positive	234	9	243
	Negative	45	218	263
Modified method	Positive	259	6	265
	Negative	20	221	241

TTE: transthoracic echocardiography; TEE: transesophageal echocardiography; PFO: patent foramen ovale.

**Table 3:** Semiquantitative shunt grading using traditional and modified Valsalva maneuver.

	Traditional Valsalva maneuver	Modified Valsalva maneuver
Negative	263(51.98%)	241(47.63%)
Positive	243(48.02%)	265(52.37%)
Mild	59(11.66%)	42(8.3%)
Moderate	47(9.29%)	64(12.65%)
Extensive	137(27.07%)	159(31.42%)

## Data Availability

The data used to support the findings of this study are available from the corresponding author upon request.
